# Inferior mesenteric arteriovenous fistula: Progress in diagnosis and management

**DOI:** 10.1097/MD.0000000000045228

**Published:** 2025-10-31

**Authors:** Congcong Shi, Lihong Chen, Ziyou Zhong, Jing Chen, Zhijian Gong, Jiahao Huang, Yufeng Xing

**Affiliations:** a The Fourth Clinical Medicine School of Guangzhou University of Chinese Medicine, Shenzhen, China; b The First Clinical Medicine School of Guangzhou University of Chinese Medicine, Guangzhou, China; c Shenzhen Traditional Chinese Medicine Hospital, Shenzhen, China.

**Keywords:** arteriovenous fistula, diagnosis, inferior mesenteric artery, management

## Abstract

Inferior mesenteric arteriovenous fistula (IMAV AVF) is a rare condition involving the inferior mesenteric artery (IMA) and inferior mesenteric vein (IMV), characterized by direct arteriovenous communication. There have been no more than 50 reported cases in the literature. The condition is categorized as congenital or acquired, with unclear pathogenesis for congenital IMAV AVF and a strong association with abdominal injuries for acquired IMAV AVF. Symptoms typically include abdominal pain, gastrointestinal bleeding, and abdominal masses. Digital subtraction angiography is considered the “gold standard” for diagnosis, revealing clusters and nodules of malformed blood vessels, thickened and increased arteries, and prematurely visible and dilated draining veins. Treatment is based on effectively eliminating or relieving venous hypertension and may involve endovascular techniques or surgery, depending on the patient’s condition. This article provides a comprehensive review of the epidemiology, etiology, pathogenesis, pathophysiology, clinical manifestations, imaging performance, differential diagnosis, and treatments of IMAV AVF to enhance clinical awareness and minimize clinical underdiagnosis and misdiagnosis.

## 1. Introduction

Arteriovenous fistula (AVF) is characterized by direct communication between arteries and veins.^[1]^ AVF can be either congenital or acquired, with cases involving the inferior mesenteric artery (IMA) and inferior mesenteric vein (IMV) being particularly rare and referred to as Inferior mesenteric arteriovenous fistula (IMAV AVF). No more than 50 cases have been reported in the literature.^[2]^

IMAV AVF often presents symptoms such as flank pain, flank masses, and gastrointestinal (GI) bleeding. Imaging examinations typically reveal discontinuous bowel alignment. Some patients may experience mild or nonspecific symptoms that both patients and physicians easily overlook. In severe cases, IMAV AVF can lead to heart failure.^[3]^ Therefore, this paper provides a comprehensive summary of the disease based on previous literature in order to serve as a reference for the clinical diagnosis and treatment of IMAV AVF.

**Figure 1.**
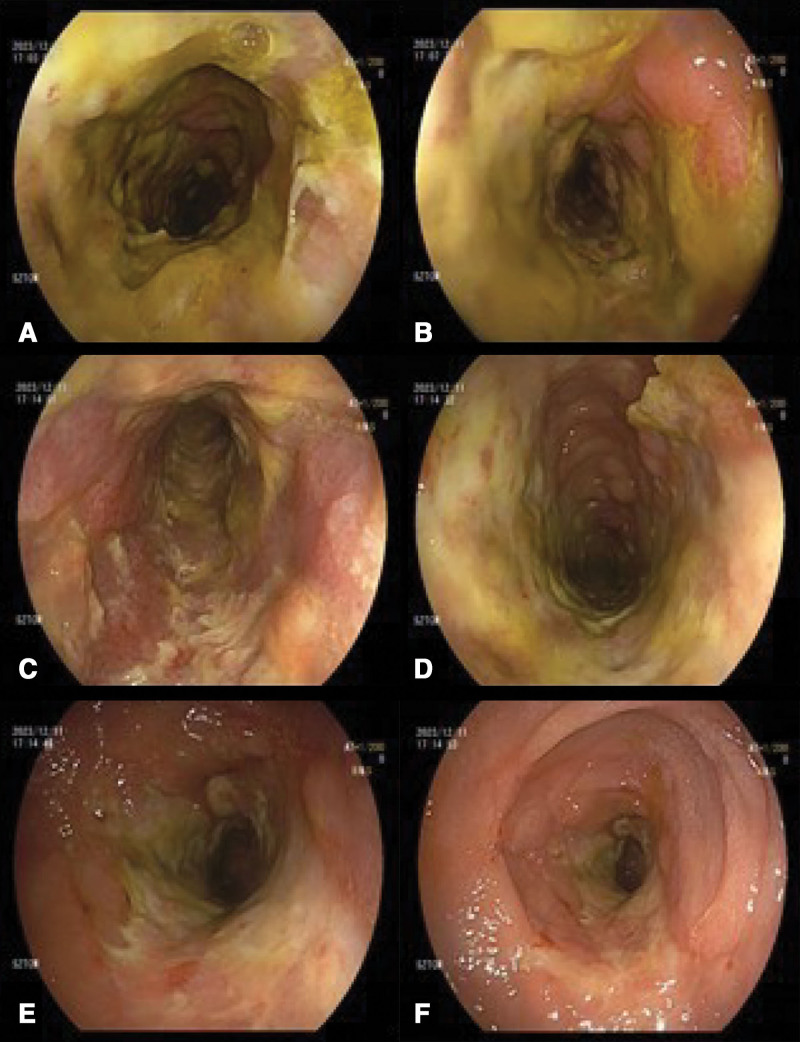
Ischemic enteritis and stenosis were observed from the sigmoid-descending colon junction to the upper rectum. Colonoscopy finding. Limited scope advancement to 50 cm from the anal verge shows abnormal colonic mucosa with multiple ulcer, exudate, hemorrhage and stenosis from the left colon to the sigmoid colon. (A–D) Left colon. (E and F) Sigmoid colon.

**Figure 2.**
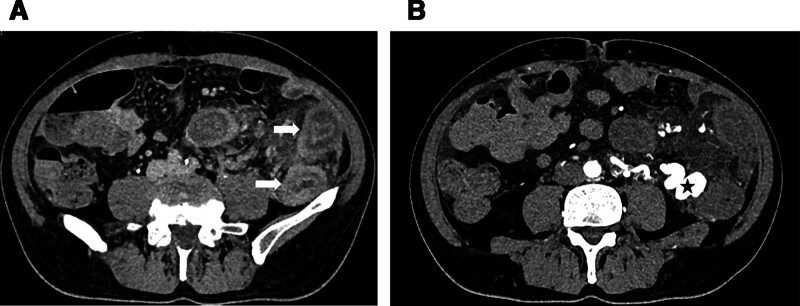
(A) Extensive intestinal wall thickening in the inferior mesenteric artery (IMA) region (arrow). (B) Hyperplasia dilatation of blood vessels (star) supplied by IMA from the sigmoid-descending colon junction to the upper rectum.

**Figure 3.**
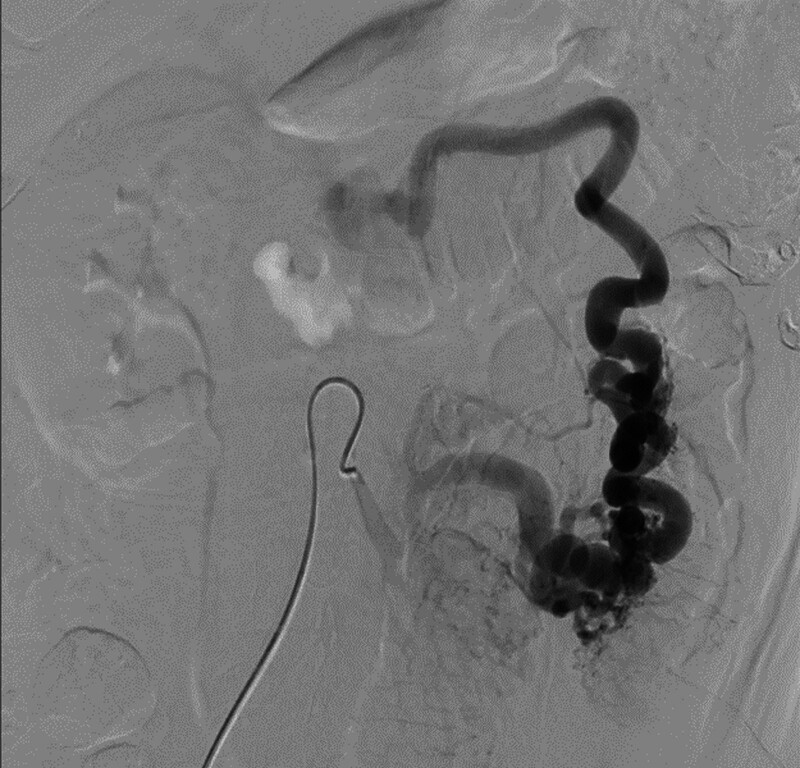
Arteriography showing large diameter and high-flow vessels.

**Figure 4.**
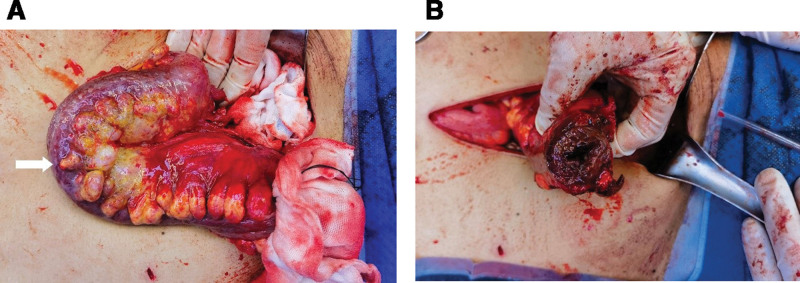
(A) Intraoperative finding of tortuous dilated arteriovenous malformation involving mainly the wall of left colon and splenic flexure (arrow). (B) Intestinal wall and mesenteric thickening of the sigmoid colon junction to upper rectum.

We evaluated a male patient who presented with 2 months of bloody, watery diarrhea, accompanied by lower abdominal pain and significant weight loss. A diagnosis of ischemic colitis (IC) was confirmed by colonoscopy, which revealed diffuse ulcerations and stenosis from the junction of the sigmoid and descending colon to the upper rectum (Fig. 1). To establish a definitive diagnosis, multidetector computed tomography (MDCT) angiography of the abdominal aorta, superior mesenteric artery, and IMA was performed (Fig. 2). Angiography identified a nidus of an IMA to inferior mesenteric vein arteriovenous fistula (IMAV AVF) originating from the IMA and draining into the IMV (Fig. 3). To achieve complete resection of the arteriovenous malformation (AVM), we performed a resection of the descending and sigmoid colon following a 15 cm midline laparotomy incision (Fig. 4). Pathological examination confirmed the presence of IC and the IMAV AVF. A colostomy was subsequently created. On the eighth postoperative day, the patient was discharged from the hospital after an uneventful recovery. Follow-up colonoscopies and computed tomography (CT) scans conducted over the nearly 1-year postoperative period revealed no abnormalities.

## 2. Etiology

The etiology of IMAV AVF may be congenital or acquired by other conditions. Congenital lesions may be associated with undegenerated embryonic blood vessels or collagen defects.^[4]^ The disease may result from vascular malformations caused by developmental arrests or abnormalities during any or all periods of embryonic development of the circulatory system. These abnormalities during the vascular basal stem formation stage allow the persistence of abnormal vascular lumina and the formation of larger AVFs, also known as AVMs in some literature.^[5]^ Acquired lesions involving penetrating trauma,^[6]^ abdominal surgery,^[7]^ or other factors, such as Arterial cannulation, cholangiography, splenic portal venography,^[8]^ also play an essential role in the formation of AVMs.

## 3. Pathophysiology

Physiologically, the primitive aorta evolves into segmental abdominal arteries, which serve as precursors to the 3 major mesenteric vessels. This ultimately led to the development of the IMA from segmental 22 arteries. Conversely, the IMV runs between the left colonic artery and the sigmoid artery that emanates from the IMA.^[9]^ The IMA is a tributary branch of the abdominal aorta, while the IMV is part of the inferior vena cava system, both playing vital roles in the body’s circulation.

AVF refers to an abnormal direct high-flow communication between an artery and a vein, bypassing the capillary bed. These fistulas can be categorized as direct or indirect. In the case of direct fistulas, neighboring arteries, and veins are simultaneously injured with wound margins directly aligned, allowing for immediate direct flow. On the other hand, the presence of a hematoma between the injured arteries and veins, or the development of a sac or tube through a disorganized vascular network, leads to the formation of an indirect fistula.^[10]^ AVFs are also present in the mesenteric vascular system, with IMAV AVF potentially causing hemodynamic changes that result in symptoms such as portal hypertension, mesenteric ischemia, and heart failure.^[11–13]^

## 4. Clinical manifestations

The presentation of symptoms varies among patients, with some exhibiting mild self-conscious symptoms and others predominantly experiencing GI symptoms such as flank pain, blood in the stool, diarrhea, constipation, and bloating.^[6,14–16]^ Additional symptoms may include suprapubic pain, flank mass, and, in severe cases, shock and respiratory distress.^[17–19]^ These clinical manifestations may be attributed to ischemic, congestive, or hemodynamic mechanisms. They can be further characterized by the presence of an abdominal mass or lump, often accompanied by a tremo.^[18]^

IMAV AVF significantly impacts hemodynamics and can lead to the development of complications such as esophageal varices, IC, ischemia of the sigmoid colon, edema of the sigmoid colon mucosa, luminal stenosis, retroperitoneal hematoma, severe portal hypertension, and cardiomyopathy, which can be reflected in abnormal biochemical indices of the corresponding organs.^[4]^

## 5. Imaging performance

Digital subtraction angiography (DSA) is considered the “gold standard” for diagnosing AVF as it can accurately determine the location and extent of the involved blood vessels. CT, CT angiography (CTA), and GI endoscopy are also used as initial screening tools to indicate the possibility of vascular lesions. Additionally, abdominal ultrasound and X-ray abdominal plain film help diagnose this disease.

### 5.1. Computed tomography

Based on high and low tissue density imaging, abdominal CT is performed as a single plain sequence to screen for possible vascular fistulas between the IMV and IMA. It shows changes in the shape of the involved bowel and the appearance of the AVF.^[3,7,15,20–22]^ Contrast-enhanced CT can examine the blood supply to the intestinal canal, the source of bleeding from the lesion, the flow direction, and the obstruction site through time-sequential scanning imaging such as venous scanning, arterial scanning, and delayed scanning.^[4]^ CTA primarily reflects vascularity and can indicate edema and wall thickening of the intestinal tubes, as well as the degree of dilatation and curvature of the involved vessels, and allows further angiography to show the supplying arteries, the draining veins and the AVF itself.^[2,7,23]^ Multidetector computed tomographic (MDCT) angiography allows for simultaneous acquisition of multiple levels of image data and can visualize the morphology of AVF as well as lesions involving specific branches of the IMA and the IMV.^[16,24]^

### 5.2. Endoscopic examination

GI endoscopy is a necessary diagnostic and therapeutic procedure in which an endoscope is inserted into the digestive tract to obtain images. In the case of IMAV AVF, colonoscopy reveals edema and congestion of the mucosa of the involved bowel and capillary dilatation, as well as multiple ulcers, exudation, and bleeding.^[2,7,24]^ GI endoscopy can also indicate the location of the diseased intestine section.^[15,16,21]^ Therefore, most clinical diagnoses are colitis and colonic ulcers. Pathologic tissue biopsy can also be done endoscopically, which is generally consistent with the features of ischemic bowel disease, such as hemorrhage and edema of the colonic mucosa and submucosa, partial necrosis and ulceration of the mucosa,^[14]^ and helps to make a differential diagnosis with inflammatory bowel disease (IBD) and intestinal malignant lesions.

### 5.3. Digital subtraction angiography

IMAV AVFs appear as clustered and nodular malformed vascular masses, thickened and increased blood-supplying arteries, and prematurely visible and dilated draining veins in DSA examination. DSA can identify high blood flow points between the IMA and IMV branches and with the hemorrhoidal plexus and define the location and extent of sinus tracts.^[4,7,16,23]^ DSA can also show the source of blood supply, direction of drainage, converging vessels, and shunt points of AVF,^[10,15,20,21,24]^ as well as the dilatation, stenosis, and occlusion of the affected vessels.^[7]^ In conclusion, DSA can further define the AVF lesion site, scope, number of involved vessels, and tube size, which can lay the foundation for further embolization and surgical treatment.

## 6. Differential diagnosis

Arteriovenous fistula can be challenging to differentiate from other conditions due to similar clinical manifestations, signs, and imaging performance. These conditions include arteriovenous malformation (AVM), hemangioma, pseudoaneurysm, and IBD.

### 6.1. Arteriovenous malformation (AVM)

AVM is characterized by abnormal vascular channels with feeding arteries and draining veins but without a regular intervening capillary network. Except for rare high-flow lesions, the majority of patients are asymptomatic. Physical examination may reveal local erythema, hyperthermia, prominent pulsations, a palpable thrill, and an audible bruit.^[25]^ High-resolution imaging such as CTA or magnetic resonance angiography imaging (MRA) can help distinguish AVM from AVFs.^[26]^ However, it is essential to note that in some literature, congenital AVF is also referred to as AVM.^[5]^

### 6.2. Hemangioma

Hemangiomas, commonly known as “strawberry scars,” are the most common benign tumors in infancy caused by the proliferation of endothelial cells. It can be categorized into 3 types: superficial lesions, which involve the superficial dermis and are raised, lobulated, and bright red. Deep hemangiomas, called subcutaneous hemangiomas, arise from the reticular dermis and/or the subcutis layer and appear as a bluish-hued nodule, plaque, or tumor. Mixed hemangiomas have features of both locations^.[27,28]^ They can both present with symptoms such as bleeding and can be differentiated by CTA or MRA.

### 6.3. Pseudoaneurysm

Pseudoaneurysms often occur at sites of arterial injury caused by trauma or infection; blood leaks from the injury site and is contained by a wall developed with the products of the clotting cascade. The most superficial pseudoaneurysms present as painful pulsatile masses, and the most common sites include the heart, femoral artery, viscera, and aorta.^[29]^ Generally, the 2 can be differentiated by abdominal ultrasound.

### 6.4. Inflammatory bowel disease

IBD, including body disease and ulcerative colitis, is characterized by chronic recurrent intestinal inflammation that involves a complex interplay between genetic, environmental, or microbial factors and the immune response. Crohn’s disease can cause transmural inflammation affecting any part of the gastrointestinal tract (most commonly the terminal ileum or the perianal region), usually with complications such as abscesses, fistulas, and strictures, and mostly with watery diarrhea and vague symptoms. In contrast, ulcerative colitis is usually characterized by mucosal inflammation and is confined to the colon, often presenting with diarrhea and bleeding.^[30,31]^ The clinical presentation of IMAV AVF and IBD is similar, but the pathologic manifestations are different and can be differentiated by further refinement of CTA and DSA.

## 7. Treatments

Most AVFs typically exhibit an abnormal vascular mass between the artery supplying the vein, referred to as the “nidus,” which consists of a dispersed network of dense, tortuous, poorly differentiated vessels with low vascular resistance. These vessels invasively recruit collateral vessels and promote vascularization. Therefore, the primary principle of treating this condition is to effectively eliminate or alleviate venous hypertension, eliminate the “nidus,” and increase distal capillary blood flow while reducing the tendency to arterialize the venous system.

### 7.1. Endovascular techniques

Endovascular techniques are widely acknowledged as the first-line treatment for AVF. In previous case reports, interventional treatment using stenting or embolization is commonly utilized for IMAV AVF with a clear fistula and feeding vessel. This approach precludes surgical removal, is less invasive, and is relatively safe.^[2,16,22]^ The primary methods of endovascular embolization to access the malformed vascular mass include transarterial, transvenous, and percutaneous approaches.^[32]^ However, these interventions may also result in organ ischemia or recurrence, particularly when more than 1 blood supply vessel is involved. The principal postoperative complications include the potential migration of embolic material when the diameter of the IMAV AVF exceeds 8 mm, and the flow rate is high, as well as varicose vein rupture due to excessive varicose pressure near the shunt vessel.^[33]^

### 7.2. Surgery

In treating complex fistulas and severe symptoms of colonic ischemia, surgical intervention is often required for certain patients. Laparotomy becomes necessary when the diameter of the visceral and exceeds 8 to 9 mm due to the risk of distal migration of the embolic agent.^[23]^ Conditions such as high blood flow and blood-supplying vessels with the risk of organ ischemia, extensive inflammation, and thickening of the mesentery of the left colon may necessitate open surgery and the creation of a terminal colostomy.^[24]^ Surgical treatment is feasible for U-shaped fistulas directly connected to the colon to reduce the risk of colonic ischemia.^[18]^ In contrast, others opt for laparoscopic sigmoidectomy to address AVF and associated varicose veins, given the high risk and low efficacy of endoscopic and radiation therapy.^[16]^

Furthermore, in cases where sub mesenteric arteriovenous fistulae are numerous, long, tortuous, and challenging to access with a catheter, they may only be treated with surgical resection.^[7]^ If embolization proves ineffective, further surgical treatment may be necessary.^[6]^ Surgical interventions are often staged, such as performing a left hemicolectomy followed by a right colorectal-rectal anastomosis 3 to 6 months later, and a colostomy 6 months later, followed by a left hemicolectomy and a right colorectal-rectal anastomosis.

In some complex cases, a combination of embolization and surgery may be used, such as in the case of rectal and gastric cancer combined with IMA portal vein fistula. The AVF of the inflow vessels can be targeted for radiological embolization, followed by staged low anterior rectal resection and gastrectomy to reduce portal vein pressure.^[33]^ Combined treatments based on different etiologies can also be considered, such as percutaneous angioplasty for portal vein stenosis and surgical repair of IMAV AVF with severe ischemic changes in the sigmoid colon.^[14]^

### 7.3. Traditional Chinese medicine (TCM)

In patients with IMAV AVF who present with minor hematochezia and no systemic symptoms, conservative treatment could be considered.^[34]^ According to TCM, congenital IMAV AVF is primarily associated with a kidney qi (shen qi) deficiency, whereas acquired IMAV AVF is mainly caused by exposure to external evils or damage to gold blades. TCM treatment offers significant advantages, as the disease is typically classified as “Chang Pi Xia Xue,” “Abdominal Pain,” or “Li Ji” based on the 4 diagnoses and references in the guiding ideology. The guiding principle of diagnosis and treatment involves clearing heat and cooling blood, promoting blood circulation and stopping bleeding, and other treatment modalities. This may include the use of herbal formulas like Pulsatilla Decoction,^[35]^ Lizhong decoction,^[36]^ and Shaoyao Decoction,^[37]^ as well as complementary treatments such as acupuncture,^[38]^ therapeutic massage,^[39]^ and retention enema with TCM.^[40]^ TCM tonics or physiotherapy may facilitate faster healing even after interventional embolization or surgery.

## 8. Conclusion

IMAV AVF is a rare condition with nonspecific clinical manifestations, making it prone to underdiagnosis and misdiagnosis. Therefore, suspicion of IMAV AVF should be raised in patients presenting with symptoms such as gastrointestinal bleeding, black stools, or hemorrhoids after common causes have been ruled out. The resemblance of IMAV AVF to an autoimmune disease and the unclear triggering mechanism suggests the possibility of an inflammatory process leading to tissue damage. Further studies could explore the potential relationship between IMAV AVF and autoimmune diseases and identify specific serum markers for the condition. Treatment of IMAV AVF typically involves endovascular techniques or surgery to target the lesion with precision. However, in cases where conservative treatment is suitable but discomfort persists during the perioperative period, TCM methods can be used as adjunctive therapy. Published literature on the combined diagnosis and treatment of IMAV AVF using TCM and Western medicine is scarce. With patient consent, retaining pathological specimens for analysis can further elucidate the etiology of the disease. In cases where patients succumb to the disease, obtaining consent from their families and ethical associations for autopsies can provide valuable insights into the pathogenesis, pathological changes, and causes of death, thereby contributing to the future diagnosis and treatment of the disease.

## Author contributions

**Conceptualization:** Congcong Shi, Yufeng Xing.

**Data curation:** Zhijian Gong.

**Formal analysis:** Yufeng Xing.

**Investigation:** Lihong Chen.

**Methodology:** Yufeng Xing.

**Project administration:** Yufeng Xing.

**Resources:** Jiahao Huang.

**Validation:** Jing Chen.

**Visualization:** Yufeng Xing.

**Writing – original draft:** Congcong Shi.

**Writing – review & editing:** Ziyou Zhong, Yufeng Xing.
